# Correspondence on “Diabetic ketoacidosis shortly after COVID‐19 vaccination in a non‐small‐cell lung cancer patient”

**DOI:** 10.1111/1759-7714.14371

**Published:** 2022-03-07

**Authors:** Rujittika Mungmunpuntipantip, Viroj Wiwanitkit

**Affiliations:** ^1^ Private Academic Consultant Bangkok Thailand; ^2^ Honorary professor, Dr DY Patil University Pune India

Dear Editor

We would like to share ideas on the publication “Diabetic ketoacidosis shortly after COVID‐19 vaccination in a non‐small ‐cell lung cancer patient receiving combination of PD‐1 and CTLA‐4 inhibitors: A case report.^1^” Makiguchi et al. stated: “We speculate that the immune‐related adverse event and immunogenicity of vaccination synergistically induced DKA.^1^” We accept that the COVID‐19 vaccination may have side effects. Sasaki et al. presented an intriguing underlying pathomechanism in which the vaccine could cause aberrant immunity and DKA.^2^ However, there is a lengthy time between vaccination and the onset of diabetes. We should be aware of the likelihood of a medical issue co‐occurring with the COVID‐19 vaccine that can lead to type 1 diabetes in a COVID‐19 vaccine recipient. A vaccination recipient, for example, could develop dengue,^3^ and dengue could be a cause of DKA.^4^ Finally, we agree with the conclusions of Sasaki et al.^2^ on the importance of a thorough assessment of the problem and the recognition of probable vaccination‐induced diabetes. On the other hand, we would like to add a note about another possible concurrent issue that could cause DKA in a vaccine recipient.

## CONFLICT OF INTEREST

The authors confirm that there are no conflicts of interest.
